# Distinguishing IDH mutation status in gliomas using FTIR-ATR spectra of peripheral blood plasma indicating clear traces of protein amyloid aggregation

**DOI:** 10.1186/s12885-024-11970-y

**Published:** 2024-02-16

**Authors:** Saiko Kino, Masayuki Kanamori, Yoshiteru Shimoda, Kuniyasu Niizuma, Hidenori Endo, Yuji Matsuura

**Affiliations:** 1 Graduate School of Biomedical Engineering, Tohoku University, 6-6-05, Aza-Aoba, Aramaki, Aoba, Sendai City, 980-8579 Miyagi Prefecture Japan; 2 Department of Neurosurgery, Tohoku University Graduate School of Medicine, 980-8574 Seiryo 1-1, Aoba, Sendai City, Miyagi Prefecture Japan; 3 Department of Neurosurgical Engineering and Translational Neuroscience, Graduate School of Biomedical Engineering, Tohoku University, Seiryo 2-1, Aoba, Sendai City, 980-8575 Miyagi Prefecture Japan; 4 Department of Neurosurgical Engineering and Translational Neuroscience, Tohoku University Graduate School of Medicine, 980-8575 Seiryo 2-1, Aoba, Sendai City, Miyagi Prefecture Japan

**Keywords:** Glioma, IDH mutation, Blood biomarkers, Protein aggregation, Amyloid, Mid-infrared absorption spectroscopy

## Abstract

**Background:**

Glioma is a primary brain tumor and the assessment of its molecular profile in a minimally invasive manner is important in determining treatment strategies. Among the molecular abnormalities of gliomas, mutations in the isocitrate dehydrogenase (IDH) gene are strong predictors of treatment sensitivity and prognosis. In this study, we attempted to non-invasively diagnose glioma development and the presence of IDH mutations using multivariate analysis of the plasma mid-infrared absorption spectra for a comprehensive and sensitive view of changes in blood components associated with the disease and genetic mutations. These component changes are discussed in terms of absorption wavenumbers that contribute to differentiation.

**Methods:**

Plasma samples were collected at our institutes from 84 patients with glioma (13 oligodendrogliomas, 17 IDH-mutant astrocytoma, 7 IDH wild-type diffuse glioma, and 47 glioblastomas) before treatment initiation and 72 healthy participants. FTIR-ATR spectra were obtained for each plasma sample, and PLS discriminant analysis was performed using the absorbance of each wavenumber in the fingerprint region of biomolecules as the explanatory variable. This data was used to distinguish patients with glioma from healthy participants and diagnose the presence of IDH mutations.

**Results:**

The derived classification algorithm distinguished the patients with glioma from healthy participants with 83% accuracy (area under the curve (AUC) in receiver operating characteristic (ROC) = 0.908) and diagnosed the presence of IDH mutation with 75% accuracy (AUC = 0.752 in ROC) in cross-validation using 30% of the total test data. The characteristic changes in the absorption spectra suggest an increase in the ratio of β-sheet structures in the conformational composition of blood proteins of patients with glioma. Furthermore, these changes were more pronounced in patients with IDH-mutant gliomas.

**Conclusions:**

The plasma infrared absorption spectra could be used to diagnose gliomas and the presence of IDH mutations in gliomas with a high degree of accuracy. The spectral shape of the protein absorption band showed that the ratio of β-sheet structures in blood proteins was significantly higher in patients with glioma than in healthy participants, and protein aggregation was a distinct feature in patients with glioma with IDH mutations.

## Background

Diffuse gliomas are the most malignant brain tumors. Its prognosis, optimal treatment, and response to therapy vary greatly depending on histological findings and molecular aberrations, including genetic mutations. The WHO Classification of the Central Nervous System Tumors, 5th edition, published in 2021, describes the presence or absence of isocitrate dehydrogenase (IDH) mutation as one of the essential findings in the diagnosis of adult diffuse glioma [1–9]. Adult diffuse gliomas are classified as astrocytoma, IDH-mutant; oligodendroglioma, IDH-mutant and 1p/19q co-deleted; and glioblastoma, IDH-wildtype. Diffuse gliomas with IDH mutation indicate a better prognosis than glioblastoma with wild-type IDH gene [7, 8]. In clinical practice, these classifications are confirmed postoperatively by analyzing the tumor deoxyribonucleic acid (DNA) and tissue sections; however, diagnosing the mutation status of the IDH gene before or during tumor resection is desirable to determine the suitable surgical strategies based on the molecular findings [10, 11]. To achieve this, the following techniques have been developed: magnetic resonance (MR) spectrometry to detect [3–5] 2-hydroxyglutarate (2-HG), which shows increased tissue concentration in the presence of mutation [12], radiomics based on magnetic MR images [13], rapid IDH gene mutation detection from intraoperatively removed tissue, and 2-HG detection from intraoperatively resected tissues [14]. These methods are promising diagnostic tools as they possess high sensitivity and specificity. However, issues such as the cost of imaging diagnosis, the lack of standardization of imaging methods, the limited feasibility of intraoperative diagnosis owing to the need for human resources, and time constraints need to be addressed.

Alternatively, preoperative genetic diagnosis of glioma using blood samples is now being attempted to overcome these problems. Obtaining the information on IDH mutation status in glioma from peripheral blood samples will aid in determining the prognosis and predicted course of the disease before surgery and greatly assist in making early decisions on the course of treatment including the extent of resection. Blood contains tumor-derived biomarkers such as circulating tumor cells, cell-free DNA, circulating cell-free micro ribonucleic acid, circulating extracellular vesicles or exosomes, proteins, and tumor-educating platelets. Liquid biopsies are becoming increasingly important in the diagnosis of central nervous system tumors [15]. Several studies have reported attempts to identify and grade gliomas based on the analysis of nonspecific molecular and cellular components in a patient's blood such as glial fibrillary acidic protein, other proteins, and neutrophil/lymphocyte ratios [16–19]. Similarly, the identification of IDH mutations based on circulating DNA [20], and the usefulness of hydroxy methylome analysis of circulating DNA in plasma [21] have also been reported. Each of these methods, which focus on specific components, require different sample-processing methods and reagents, and costs for each component. In contrast, the recent trend employs omics technology that comprehensively analyzes information on all molecules in the body. Particularly, Fourier transform infrared (FTIR) spectroscopy is useful for analyzing all the molecular information from blood and capturing changes in multiple components in real time. Mid-infrared (MIR) region used in FTIR is called the fingerprint region because it corresponds to the fundamental vibrational energy of intramolecular bonds, and its absorption spectrum reflects the states and existence ratios of the contained substances with high sensitivity [22, 23]. Nevertheless, the complex superposition of a wide variety of biomolecules is inevitable, and the introduction of multivariate analysis is essential for interpretation. Several studies have reported the combined use of liquid biopsy with MIR spectroscopy and multivariate analysis [24, 25] such as in the simultaneous prediction of urea/glucose levels, which indicates malaria based on the severity of the IR absorption spectrum of whole blood [26], and the prediction of hepatitis B and C viral infections [27] and incidence of acquired immunodeficiency syndrome [28] based on the serum absorption spectra. In the case of tumors, disease status, and morbidity have been identified based on the IR absorption spectrum analysis of serum or plasma for lung cancer [29], brain tumors [30–40], gastric and colon cancer [41, 42], and breast cancer [43]; the estimation of marker compounds based on the absorption bands have been the primary basis for identification. These reports indicate that this method holds excellent potential in this aspect.

Tołpa et al. [44] reported that gliomas can be diagnosed with 100% accuracy using tissue sections based on the principle of IR absorption by combining Raman spectra and machine learning. Others have reported high identification rates for gliomas when using IR absorption spectroscopy of tissue sections [45, 46]. In disease identification from blood, results based on multivariate analysis of IR absorption spectra of serum have been reported for primary screening for early detection [31–36]. Some researchers have reported the differentiation of patients with glioma based on IDH mutation status through spectroscopic methods that use Raman spectra [47, 48] and IR absorption spectra [49, 50] of the tumor tissues. Cameron et al. compared the results of the IDH mutation status deduced using the IR absorption spectrum of blood with those of tissues [50] and showed that serum containing only low molecular weight components of ≤ 3 kDa after centrifugal filtration showed improved classification ability. Quesnel et al. also compared the results of IDH mutation differentiation between tissue and serum samples based on Raman spectra and found that serum was inferior to tissue in providing satisfactory results but the logistic regression analysis results were up to 85% accurate [51].

In summary, rapid identification from the IR absorption spectrum of blood, which is less invasive, is a promising method for preoperative or intraoperative molecular genetic classification of gliomas. However, the identification of IDH mutations from blood, which is the key to grade determination in the genetic classification of gliomas, has only been reported using the serum-based method described above [50, 51], and the differentiation ability and the underlying basis for such differentiation are insufficient. In this study, we used plasma, which is more commonly cryopreserved for retrospective studies, as sample to evaluate glioma development and the presence of IDH mutations. In addition, we increased the accuracy of spectral measurements by increasing the effective optical path length within individual samples. Our goal is to develop a rapid and accurate diagnostic method for identifying the presence or absence of IDH mutations, an important molecular genetic diagnosis of glioma, from minimally invasive blood samples. Our method may contribute to the early prediction of glioma prognosis and the selection of treatment strategy.

## Materials and methods

### Collection of human plasma samples

This study was approved by the Ethics Committee of Tohoku University School of Medicine (Ethics Committee No. 2023–1-321). After obtaining written informed consent from all participants, plasma samples were collected from 84 pretreatment patients (13 oligodendrogliomas, 17 IDH mutant astrocytoma, 7 IDH wild-type diffuse gliomas, and 47 glioblastomas) and 12 healthy collaborators. The diagnosis was made according to the WHO Classification of Central Nervous System Tumors, Fifth Edition, and all plasma samples from patients were collected preoperatively. Plasma samples from healthy Asian participants were purchased as control samples from Cosmo Bio Co., LTD. USA and BioIVT Inc., UK (30 each; total 60 cases). All samples and anonymized personal information were collected and managed for the study with donors’ consent. Table 1 shows the breakdown and background information of all samples. All plasma samples were collected using ethylenediaminetetraacetic acid (EDTA)-Na anticoagulation tubes and stored frozen below -30 °C until measurements.

**Table 1:** Classification of human plasma samples

Subtype	Grade 2	Grade 3	Grade 4	Total	M/F	Age (Median)
Oligodendroglioma IDH-mut 1p/19q codeleted	8	5	0	13	8/5	36—64 (45)
Astrocytoma IDH-mut	3	10	4	17	7/10	22—47 (38)
Diffuse glioma IDH wt	2	4	1	7	6/1	20—83 (62)
Glioblastoma IDH wt	0	0	47	47	28/19	24—77 (60)
Control	-	-	-	72	47/25	21—63 (40)
Total	13	19	52	156	96/60	20—83 (47)

*IDH* Isocitrate dehydrogenase, *mut* mutant, *wt* wild-type, *M* male, *F* female

### Measurement of FTIR-ATR spectra

**Fig. 1:**
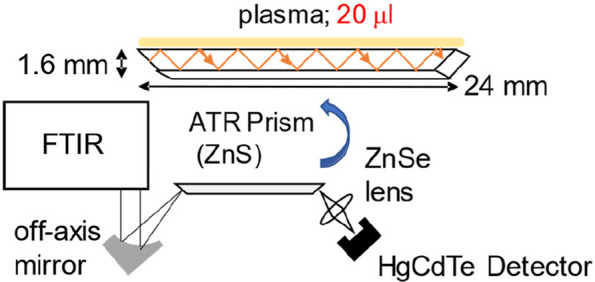
Fourier transform infrared-attenuated total reflection (FTIR-ATR) measurement system

The attenuated total reflection (ATR) method is a technique for measuring the absorption spectrum of a sample using light that marginally seeps to the side of the low refractive index region when light propagating in a medium with a high refractive index is totally reflected. In this study, a ZnS internal reflection element (IRE) with a refractive index of 2.19 in the MIR region was used. Figure 1 shows the measurement system. Infrared light emitted from an FT-IR is focused on a ZnS ATR trapezoidal IRE using an off-axis mirror, and the output beam from the IRE is collected using the HgCdTe detector. In conventional ATR measurement systems, the number of reflections at the sample/IRE interface is one to several times, however, this measurement system uses a small and long IRE (2 mm wide, 1.6 mm thick, and 24 mm long) to obtain eight reflections even with a small sample volume, which enables high sensitivity measurements.

**Fig. 2:**
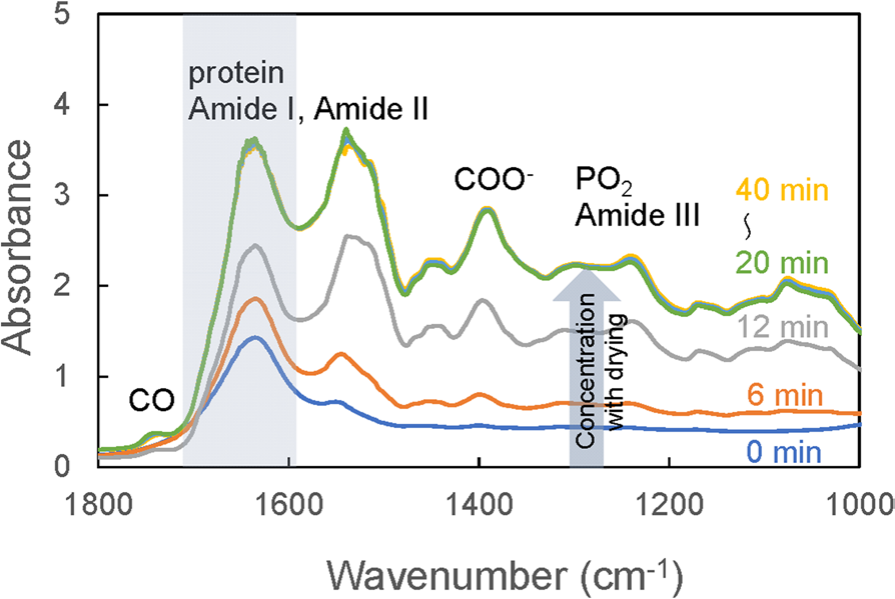
Temporal variation of infrared ATR spectra of plasma with drying. Each absorption spectrum is different in the time elapsed to start the ATR spectrum measurement after the plasma sample is applied to the top surface of the prism (blue: 0 min, orange: 6 min, gray: 12 min, green: 20 min, light blue: 30 min, yellow: 40 min). The overall absorbance increases as the sample dries (in the direction of the gray arrow); the sample dries completed in approximately 20 min and the absorbance stabilizes thereafte

Frozen plasma was thawed spontaneously at room temperature for 1 h, and 20 μL/sample was applied to the entire top surface of the IRE prism. The wavelength resolution was 4 cm^−1^, and the number of integrations was 64. Figure 2 shows the MIR absorption spectrum of a plasma sample from a healthy participant for each unit of time elapsed from application on the prism to the start of measurement. Immediately after its application on the prism (0 min), the plasma dries and concentrates with the passage of time, and the absorbance increases. In contrast, the absorption peak of water (around 1650 cm^−1^), indicated by the background color in the figure, becomes relatively small. Despite some individual differences among the samples, they dried out after approximately 20 min, and the absorbance ceased to increase thereafter. The absorption peaks corresponding to proteins, lipids, carbohydrates, urea, and other biomolecules were clearly observed in the dried plasma spectrum. The dried plasma covers the entire top surface of the prism, and a stable measurement field of view can be secured using the method of direct spontaneous drying on the prism. The absorption spectrum of dried plasma showed high reproducibility during repeated measurements of the same sample. This procedure was established as the measurement protocol in this study, and the absorption spectra of dried plasma were collected for each sample.

### Statistical analysis

Statistical analysis of all the acquired IR absorption spectra was performed using the following plug-in software**:** Lumivero (2023) XLSTAT, statistical and data analysis solution, Paris, France. https://www.xlstat.com/ja.

## Results

### Identification of glioma incidence using the plasma spectra

**Fig. 3:**
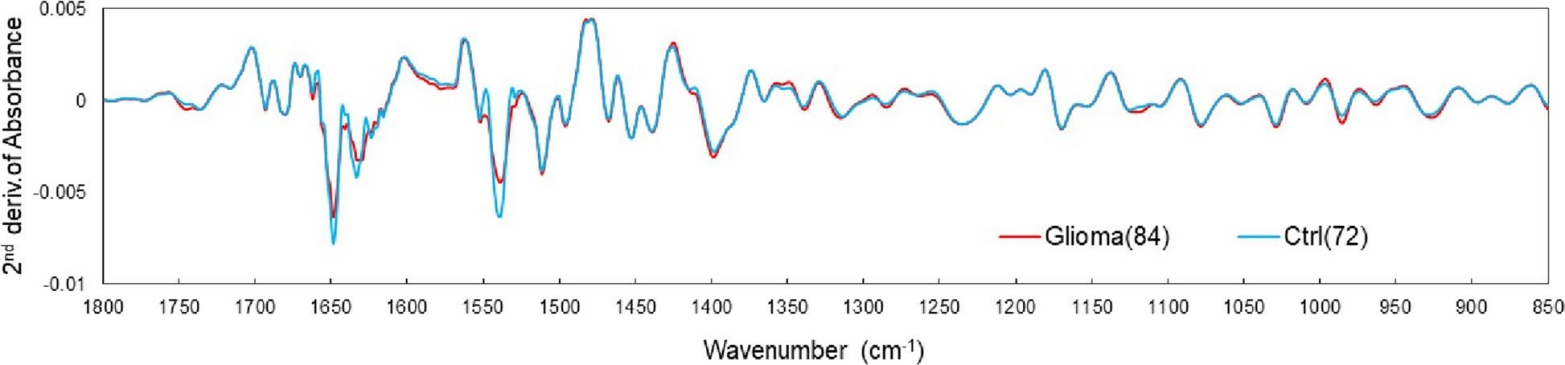
Mean values for each group in the second derivative spectrum of plasma. Glioma group (84 patients) and healthy group (72 persons)

The obtained ATR spectra were subjected to a second derivative treatment based on the Savitzky–Golay (n = 4) method. No spectra were excluded from the analysis in this study owing to outliers and other reasons. Figure 3 shows the mean values estimated from the second derivative spectra corresponding to the plasma from the glioma (84 patients) and healthy (72 persons) groups. The differences in the absorption bands between the healthy and patient groups with respect to amide I and II (1700–1500 cm^−1^) (which differ in the peptide binding of proteins) and metabolites such as lipids and sugars (1200–840 cm^−1^) were observed. The differences between the two groups were analyzed using partial least squares discriminant analysis (PLS-DA) to identify the patients with glioma based on the IR absorption spectra of plasma [52]. The PLS regression method is widely employed in multivariate analysis such as spectroscopy and is the method of choice while using explanatory variables that exceed the number of observation points. PLS-DA is a powerful discriminant method that maximizes the separation of values of each group through regression. In this study, only the absorbance at each wavenumber was used as the explanatory variable because glioma incidences were distinguished based on the absorption spectrum. The single dimensionality simplifies the assessment of the contribution to linear discrimination. Regression prediction methods may exhibit high predictive performance when using quantitative or qualitative variables with different dimensions as explanatory variables or when variables are hierarchized or networked and given correlation weights. However, it is difficult to assess the correlation among explanatory variables and their individual contributions to discrimination. Moreover, when distinguishing a small number of observation points, as in this study, the risk of overfitting is high. In this method, a spectrum with a known unique result (such as the presence or absence of disease, in this study) is used as the teacher data, and the absorbance at a certain wavenumber is used as the explanatory variable to derive a linear discriminant function such that the linear sum of the explanatory variables, which converges to a single value for the same group, whereas the values between groups are separated as much as possible (the distance between groups is maximized). For unknown spectra, the values obtained using this discriminant function (discriminant scores) are used to determine which group the spectrum falls into. The absorption wavenumber range used as an explanatory variable was set to 1800–840 cm^−1^, which is the fingerprint region of biomolecules and is less affected by prismatic atmospheres such as humidity. The absorbance values were obtained in 2 cm^−1^ increments; hence, 481 explanatory variables were used.

**Fig. 4:**
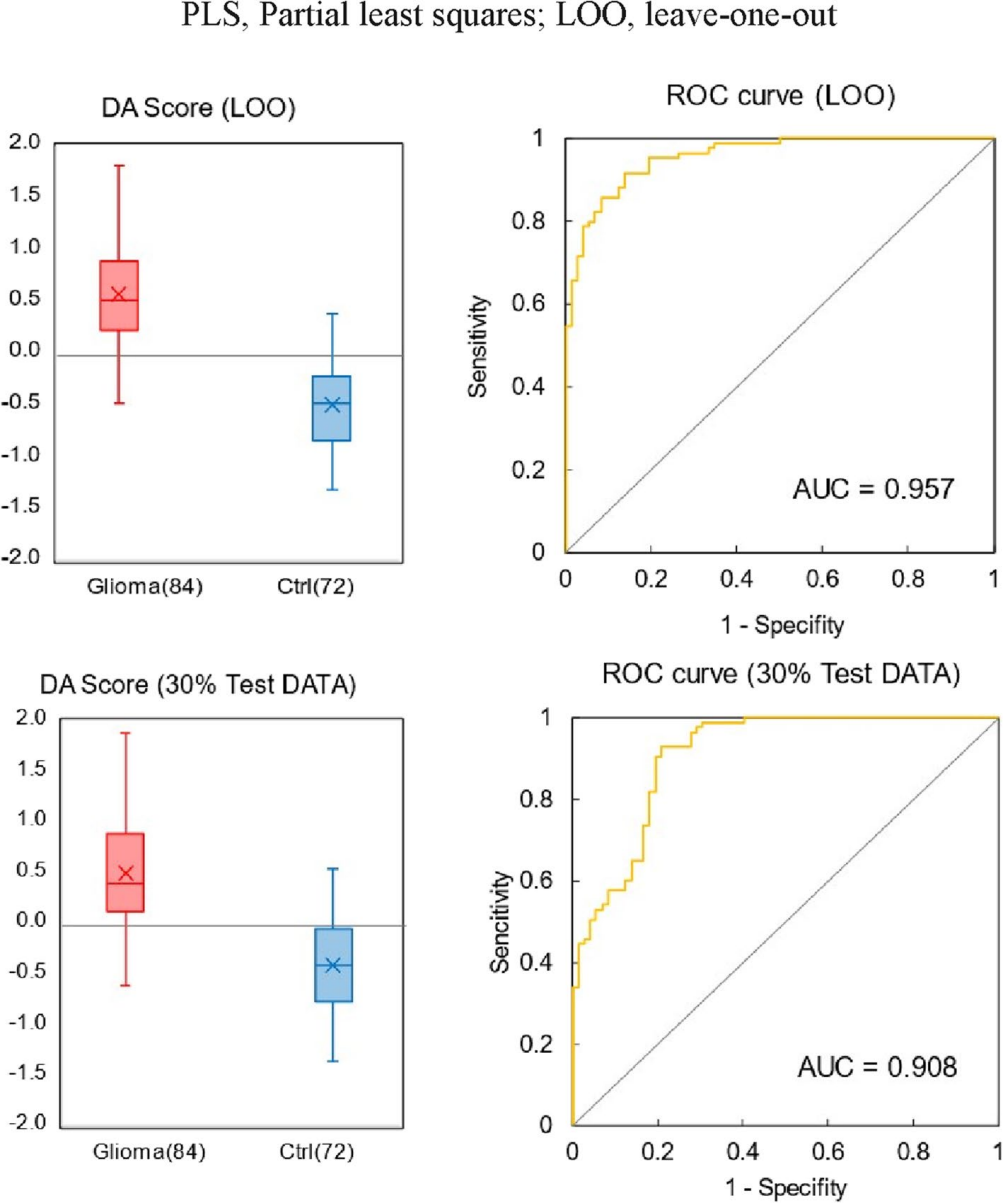
Discriminant score distribution and receiver operating characteristic (ROC) curves. For PLS discrimination of glioma and control groups from plasma infrared absorption spectra with 2 components. Cross-validation of LOO and 30% of the test data

Table 2 shows the results of the cross-validation of glioma incidence differentiation. Cross-validation was performed both in the leave-one-out (LOO) case, in which the test data were extracted one by one from the training data and in the case where the ratio of training data to test data was set to 7:3 and 30% of the test data were randomly extracted. The results for both cases are shown in Table 2. PLS regression yields varying prediction results depending on the number of components used. Increasing the number of components improves predictive performance on training data, however, surpassing the optimal number decreases predictive performance on test data. In this study, for distinguishing glioma cases, we adopted the number of components that demonstrated the highest predictive discrimination performance against test data in LOO, which was 2. The same number of components was also set to 2 in cross-validation using 30% of the test data. In the cross-validation with the 7:3 case, 10 trials were repeated with 30% of the test data randomly selected. All samples were extracted at least once during the 10 trials, and the discriminant scores derived from all extraction times were averaged for each sample. The distribution of discriminant scores and receiver operating characteristic (ROC) curves for the two groups in this cross-validation are shown in Fig. 4. The boundary of the discriminant scores in Fig. 4 is 0 points. Setting this boundary to a negative value to prevent false negatives increases the number of false positives, and as a discriminant model, sensitivity (the percentage of positive responses) increases as specificity (the percentage of negative responses) decreases. The ROC curve shows this characteristic, and the closer the area under the curve (AUC) is to 1, the higher is the discrimination accuracy (overall correct rate). The discrimination level is considered acceptable when the AUC > 0.7 [53]. The LOO case showed AUC = 0.957 and an accuracy of 89.1%; 30% of the test data showed AUC = 0.908 and an accuracy of 83.4%. The balance between sensitivity, specificity, and accuracy was maintained in the cross-validation, indicating that the IR absorption spectrum of plasma alone has sufficient potential to identify glioma incidence.

**Table 2:** PLS discrimination of glioma and control groups based on plasma infrared absorption spectra. Cross-validation of LOO and 30% of the test data

Glioma vs Ctrl	LOO	30% Test Data
Sensitivity (%)	91.7	87.0(76.7—96.6)
Specificity (%)	86.1	79.0(68.4—88.9)
Accuracy (%)	89.1	83.4(78.7—93.6)

*PLS* Partial least squares, *LOO* Leave-one-out

**Fig. 5:**
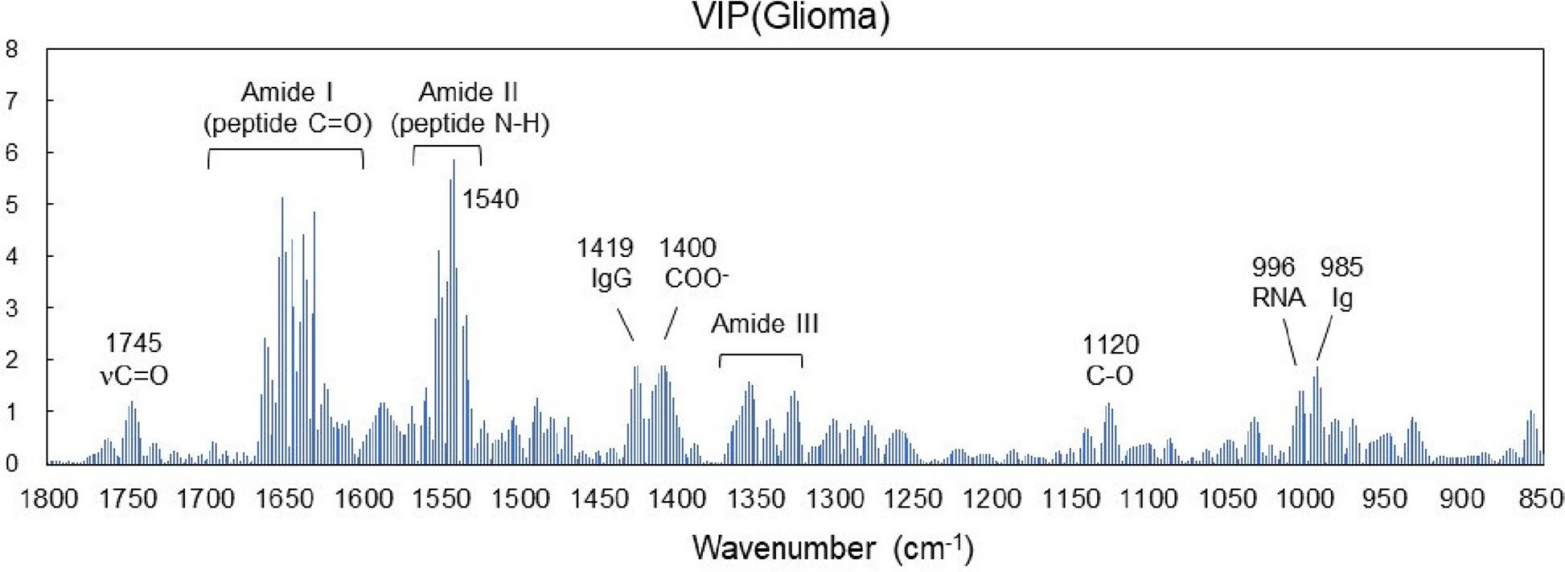
Variable importance of projection in PLS discrimination of glioma from plasma infrared absorption spectra

The variables importance of projection (VIP) in this PLS-based disease discrimination is shown in Fig. 5. In metabolomics-based biomarker search, metabolites are selected using a threshold value of VIP > 1 in a linear regression model [54]. As clearly shown in Fig. 3, the intensity of amide II (approximately 1540 cm^−1^), which exhibits N–H stretching vibration, is particularly decreased in the glioma group compared with that of the normal group, and this absorption band has the highest VIP. Apart from the amide I and II absorption bands, the VIP exceeded 1 in the absorption bands [23, 55, 56] for the antibody protein immunoglobulin (1419, 985 cm^−1^), nucleic acid (996 cm^−1^), lipid esters (1400, 1120 cm^−1^), and carbonyl (1745 cm^−1^). In addition to proteins, metabolic compounds such as nucleic acids and lipids also contributed significantly to the identification of patients with glioma based on the infrared absorption spectrum of plasma.

### Distinguishing IDH mutation status in glioma using plasma spectra

**Fig. 6:**
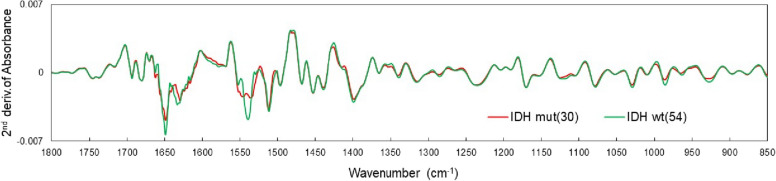
Mean values for each group in the second derivative spectrum of plasma. Isocitrate dehydrogenase (IDH)-mutant (30 patients) and IDH wild-type (54 patients) groups

Figure 6 shows the mean values of the second derivative plasma spectra of patients with IDH-mutant (30 patients) and IDH wild-type (54 patients) glioma before the start of treatment. The IDH-mutant spectra showed higher average values for amide I and II absorption bands than those in the wild-type spectra. Additional differences were observed in the absorption bands of metabolites such as lipids and sugars (1200–840 cm^−1^). The differences between the two groups were used to determine IDH mutation status in patients with glioma based on the IR absorption spectra of plasma. As in the case of disease identification, the absorbance of the second derivative spectrum in the fingerprint region of 1800–840 cm^−1^ was used as the explanatory variable for PLS-DA.

**Fig. 7:**
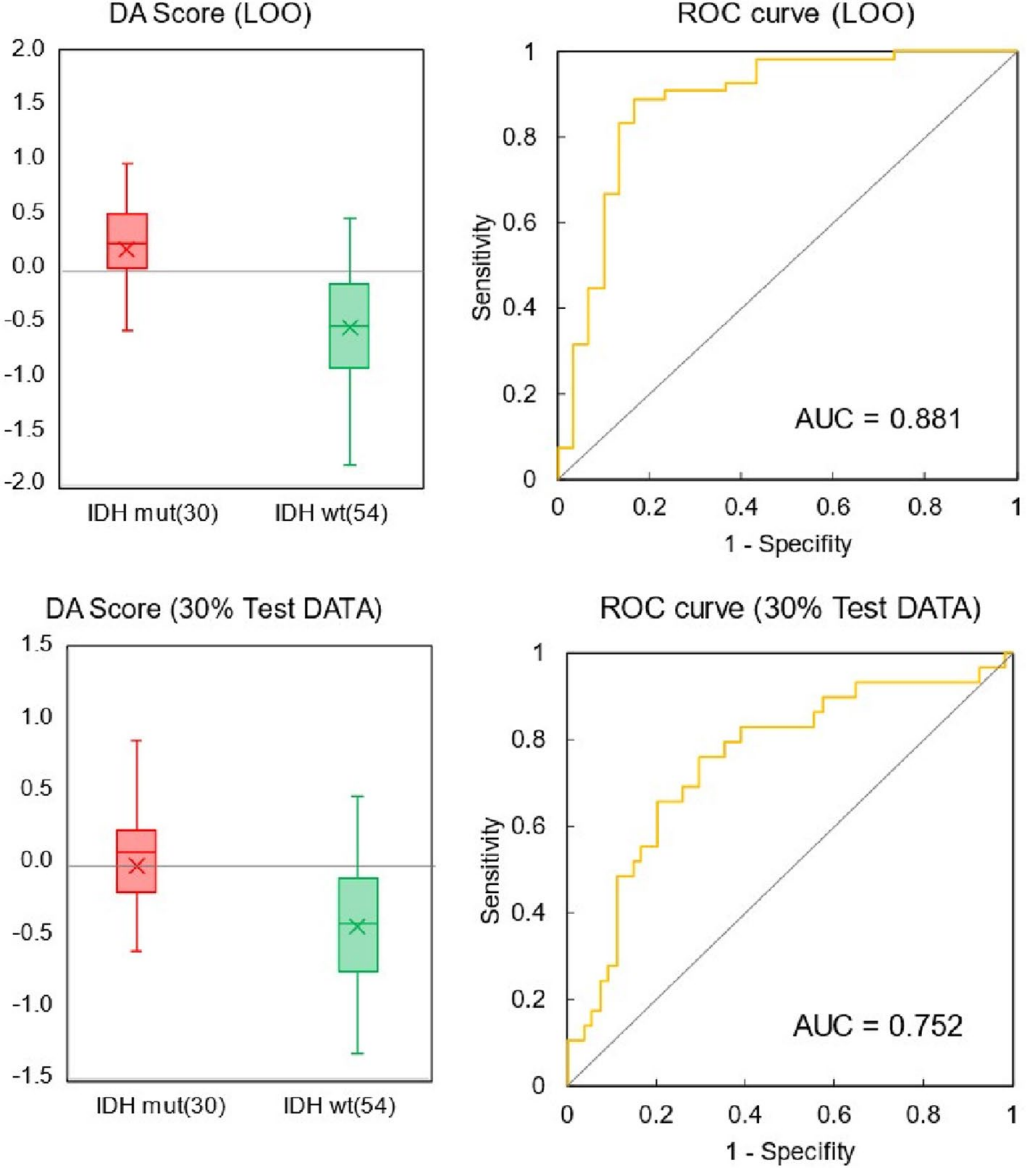
Discriminant score distribution and ROC curves. For PLS discrimination of IDH-mutant and wild-type groups from plasma infrared absorption spectra with 1 component. Cross-validation of LOO and 30% of the test data

The results of the cross-validation of this analysis are shown in Table 3. In addition to the LOO case, 10 trials were repeated with the ratio of training data and test data set to 7:3 and 30% of the test data randomly extracted. The number of components was set to 1, which had the highest predictive discrimination performance for the test data in the LOO for distinguishing IDH mutation status. The number of components for cross-validation on 30% of the test data was also set to 1. All specimens were extracted at least once in 10 trials, and the discriminant scores derived from all the extraction times were averaged for each specimen. The distribution of discriminant scores and ROC curves for the two groups in this cross-validation are shown in Fig. 7. The boundary of discriminant scores in Fig. 7 is zero. The LOO data showed AUC = 0.881 with 85.7% accuracy, and 30% of the test data showed AUC = 0.752 with 75.2% accuracy. In both cross-validation methods, the sensitivity (that is the percentage of correct answers for the mutant-type) was less than the specificity (that is the percentage of correct answers for the wild-type). This result is unavoidable given the ratio of mutant to wild type, but prior probability was not considered in this discrimination. Although further expansion of the number of cases could be improved, the 75% accuracy observed during cross-validation indicates that this method could potentially assist in diagnosis prior to tissue biopsy.

**Table 3:** PLS discrimination of IDH mutations from plasma infrared absorption spectra. Cross-validation of LOO and 30% of the test data

IDH mut vs wild	LOO	30% Test Data
Sensitivity (%)	76.7	61.4(50.0—80.0)
Specificity (%)	90.7	84.1(64.7—100.0)
Accuracy (%)	85.7	75.2(68.0—84.0)

*IDH* Isocitrate dehydrogenase, *mut* mutant, wt, wild-type; LOO, leave-one-out

**Fig. 8:**
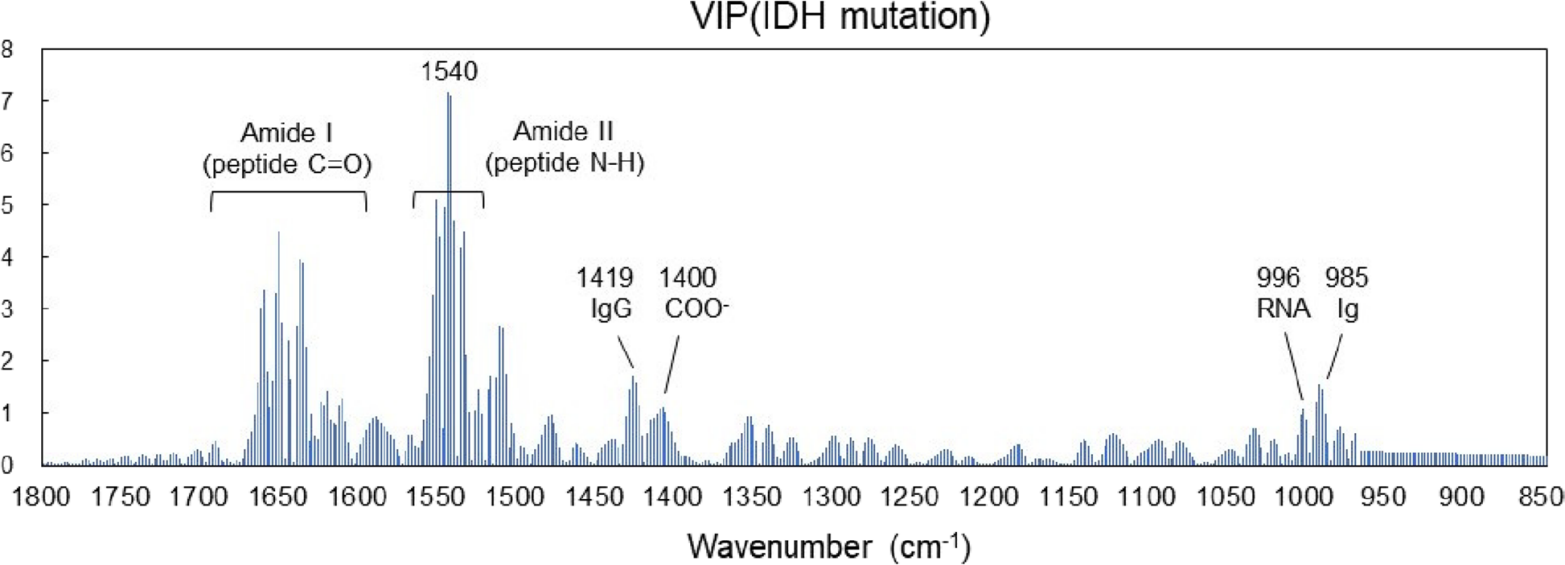
Variable importance of projection in PLS discrimination of IDH mutations from plasma infrared absorption spectra

**Fig. 9:**
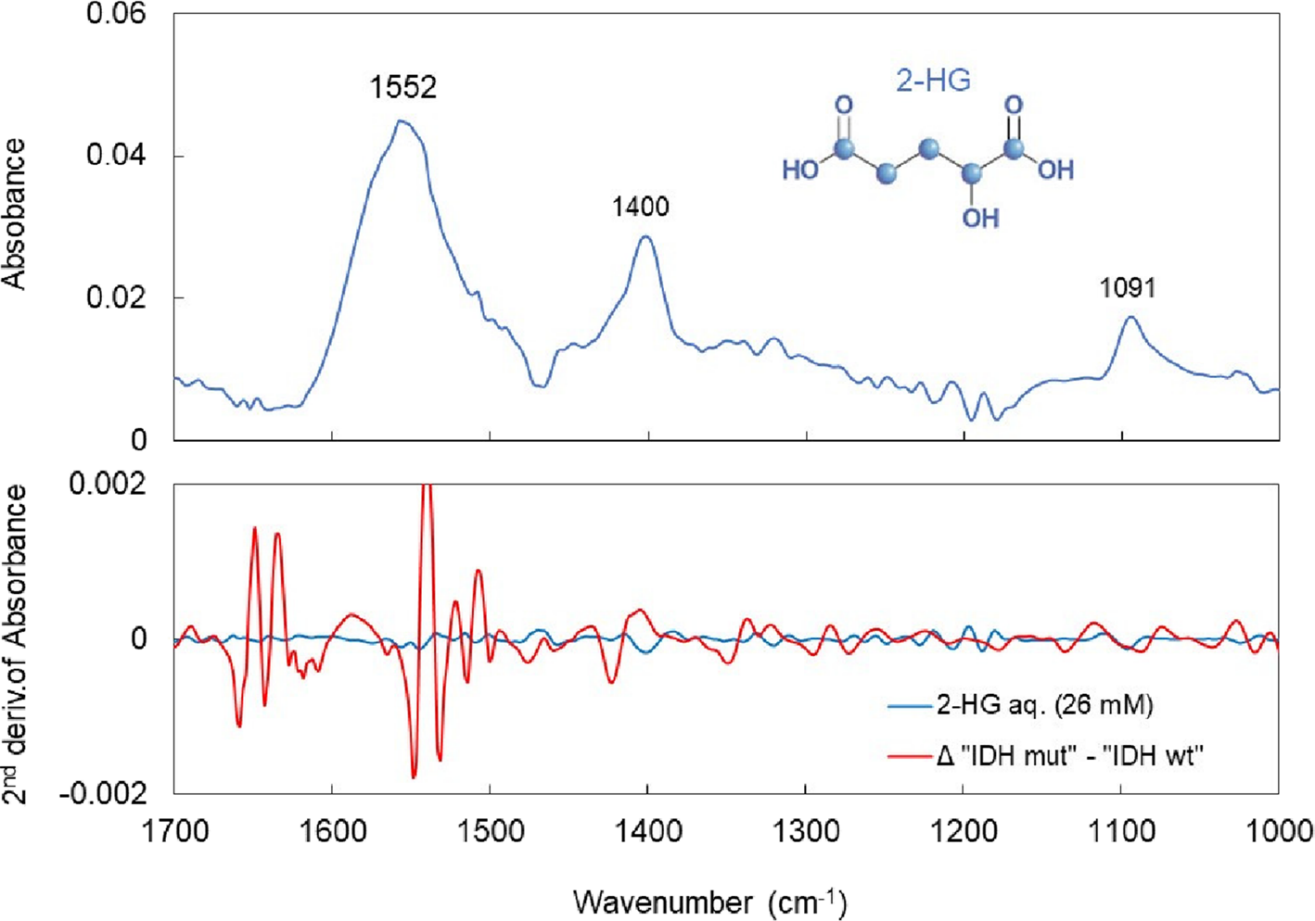
FTIR-ATR spectra of 2-hydroxyglutarate (2-HG) and differences in spectra associated with IDH mutation. The upper panel shows the FTIR-ATR spectrum of 2-HG corresponding to 0.5 wt% (26 mM), and the lower panel shows its second derivative spectrum (blue line) and the difference between the average second derivative spectra of the IDH-mutant and IDH wild-type samples in Fig. 6 (red line)

The VIP in the PLS-based identification of the IDH mutation status is shown in Fig. 8. In this case, the VIP is still significantly larger in the amide I and II absorption bands. This is because the mutant has almost no absorption around amide II (1540 cm^−1^) and the central wavenumber is shifted as shown in Fig. 6. Apart from the amide I and II absorption bands, Ig antibodies (1419 cm^−1^ and 985 cm^−1^) and nucleic acids (996 cm^−1^) contribute to VIPs similar to those in glioma disease identification (Fig. 5). In the case of IDH-mutant gliomas, 2-HG is produced in the tumor tissue and is found in the cytosol at concentrations of 5–30 mM, accumulating in the cytoplasm at high concentrations [3]. However, the difference in 2-HG levels between patients with and without the IDH mutation is the most prominent in tissue, gradually decreasing in the extracellular fluid and spinal fluid; therefore the difference is not statistically significant in the plasma [57]. The upper panel of Fig. 9 shows the FTIR-ATR spectrum of 2-HG solution (0.5% = 26 mM equivalent) measured with water as background. In the fingerprint region, absorption peaks are found at 1552 cm^−1^ (C = O), 1400 cm^−1^ (COO^−^), and 1091 cm^−1^ (C–O). However, the two peaks other than the one at 1552 cm^−1^ that is superimposed on amide II contribute little to IDH mutation differentiation. Additionally, the absorption intensity of the 1400 cm^−1^ peak is marginally lower than that of the wild type (Fig. 6). The lower panel of Fig. 9 shows the second derivative spectrum (blue line) of 2-HG at a dose equivalent to 0.5 wt% (26 mM) and the difference between the average second derivative spectra of the IDH-mutant and wild-type samples (red line) in Fig. 6. Notably, the concentration of 26 mM is close to the maximum accumulation of 2-HG in the IDH-mutant glioma tissue. However, the difference in absorption spectra between IDH-mutant and wild-type plasma is much larger than the absorption intensity of 2HG, and its spectra are distinct. These results indicate that in the plasma of glioma patients, the component that strongly contributes to the IDH mutation status is not 2-HG.

## Discussion

In this study, we aimed to establish a less invasive method for glioma diagnosis and prognosis prediction using multivariate analysis of the mid-IR absorption spectra of peripheral blood plasma. The established method distinguished between glioma patients and healthy persons with an accuracy of 83% and a cross-validation of 30%. This value is similar to those previously reported for tissues [44–46] and serum [31–36]. The absorption wavenumbers that were of high importance in differentiation were the absorption bands of amides I and II, which originate from the peptide bonds of proteins. This concurs with the assertion of Theakstone et al. [30] that the absorption bands of amides I and II are most important for differentiating patients with brain tumors based on the IR absorption spectrum of serum. Moreover, the absorption bands of immunoglobulins (1419, 985 cm^−1^), nucleic acids (996 cm^−1^), lipid esters (1400, 1120 cm^−1^), and carbonyls (1745 cm^−1^) were also of importance. Tołpa et al. reported that Raman spectra based on the infrared absorption principle distinguished tumor tissue from normal tissue [44] and that significant differences were observed between tumor and normal tissue in several Raman bands related to proteins, lipids, and nucleic acids. The results of this study indicate that the same tissue biomarkers are crucial in distinguishing patients with glioma using blood samples.

During previous instances where IDH mutation status was determined based on the IR absorption spectrum analysis of serum, Cameron et al. showed a discrimination accuracy of up to 69% in a 30% cross-validation [50] and Quesnel et al. showed an accuracy of 85% [51]. However, considering that the latter did not report any cross-validation values, the accuracy of 75% reported in this study, even with an average of 30% cross-validation, can be considered excellent. Cameron et al. were initially unable to distinguish IDH mutation status through PLS analysis based on values obtained from whole serum; however, the performance improved to an acceptable level after the high molecular weight components were removed through centrifugal filtration leaving behind the low molecular weight (≤ 3 kDa) components. Regarding the absorption wavenumbers used to distinguish IDH mutation status, the importance of protein molecules was still high among the low molecular weight components (< 3 kDa), which is consistent with our results. In this study, we analyzed plasma, which possibly imparted the same effect as that of using centrifugal filtration at the sample preparation stage. Additionally, they used a single-reflection IRE for measurement, whereas, in this study, a multi-reflection IRE was used. Multi-reflection IRE may have increased the effective optical path length in the sample and improved accuracy during FTIR-ATR measurement. The high level of measurement accuracy achieved in this study is evidenced by the fact that the statistical analysis was free of excluded spectra due to outliers. Therefore, discrimination performance improved without requiring additional processing steps on the sample.

**Fig. 10:**
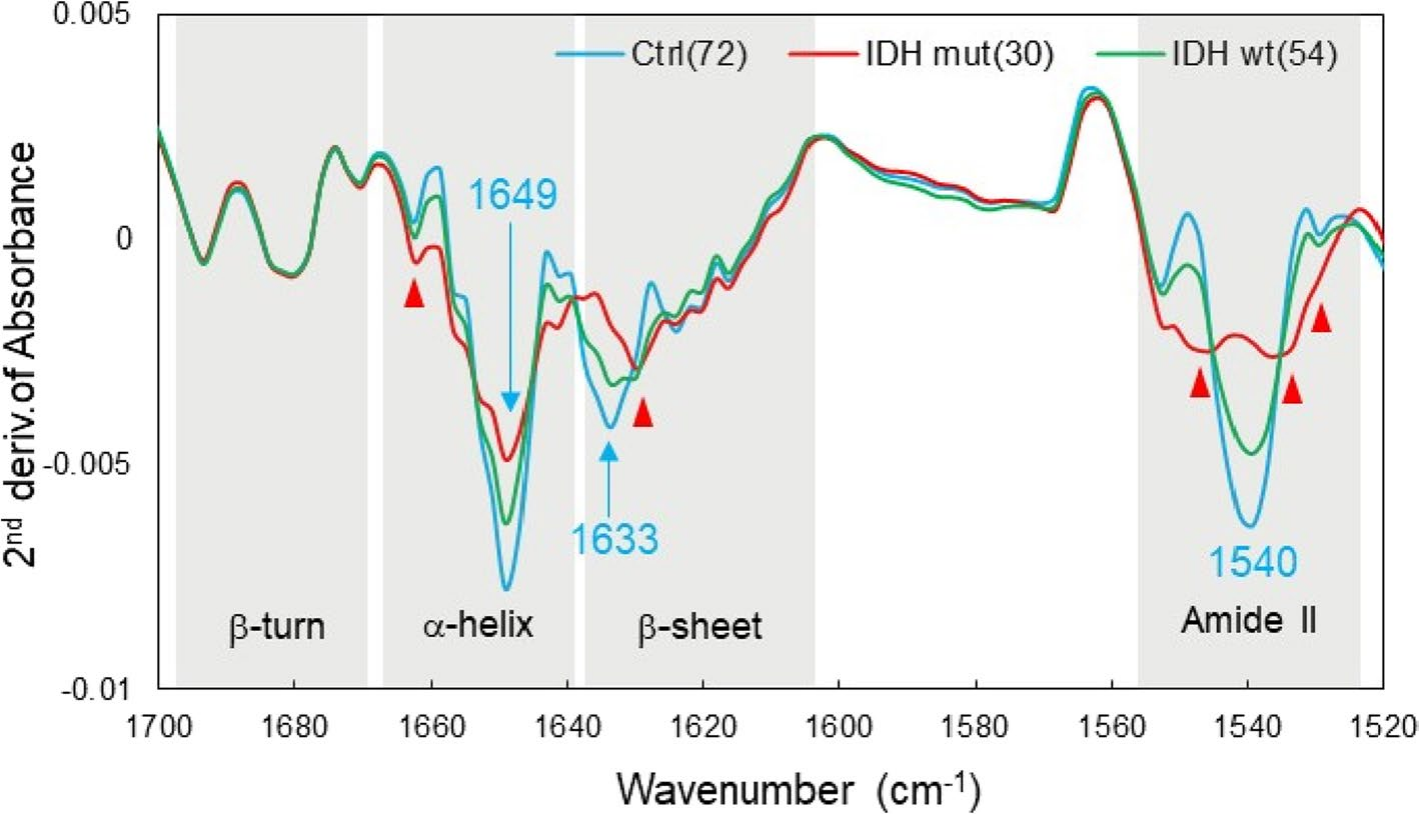
Mean values for each group in the second derivative spectrum of plasma. IDH-mutant glioma group (30 patients), IDH wild-type glioma group (54 patients), and healthy control group (72 persons)

Figure 10 shows the averaged second derivative spectra of the amide I and II regions of plasma from patients with IDH-mutant gliomas (30 patients), patients with IDH wild-type gliomas (54 patients), and healthy participants (72 persons). Deconvolution of the differential spectrum of the amide I region, which shows C = O stretching vibrations of peptide bonds, provides information about the conformational composition of the protein. First, the absorption at the center frequency of 1649 cm^−1^, indicating the α-helix structure, and at the center frequency of 1633 cm^−1^, indicating the β-sheet structure, decreased in the order of healthy, wild-type, and mutant samples. In contrast, no change was observed in the absorption intensity in the 1700–1670 cm^−1^ band, which indicates the β-turn structure. These results indicate that the β-sheet is polymerized after the transition from the α-helical structure to the β-sheet structure [58–61]. In the amide I region, the absorption wavenumbers 1662 cm^−1^ and 1629 cm^−1^ (indicated by arrows in the figure) increased in the order of normal, wild-type, and mutant samples, suggesting the presence of protein aggregates with high β-sheet content, such as amyloid beta (Aβ) [62, 63]. This phenomenon is more easily observed in the amide II region, which exhibits N–H stretching vibrations of peptide bonds. Absorption at the central wavenumber 1540 cm^−1^ decreased as the β-sheet folded in the order of normal, wild-type, and mutant samples, and conversely increased at 1549, 1534, and 1528 cm^−1^ (arrows in the figure) in the mutant sample, suggesting the formation of amyloid oligomers [59]. Amyloid is known to be elevated in many cancers, and the plasma levels of Aβ peptide are significantly higher in patients than in non-patients [64]. Similarly, in gliomas, the accumulation of Aβ has been reported in tumor tissue and the stellate cells around affected blood vessels [65]. The major targets for molecular diagnosis in gliomas are IDH, tumor suppressor protein TP53, phosphatase and tensin homolog (PTEN), telomerase reverse transcriptase (TERT), α-thalassemia/mental retardation syndrome X-linked (ATR-X), and epidermal growth factor receptor (EGFR). Several mutant proteins related to cell cycle control and apoptotic pathways have a high propensity to form amyloid [66].

**Fig. 11:**
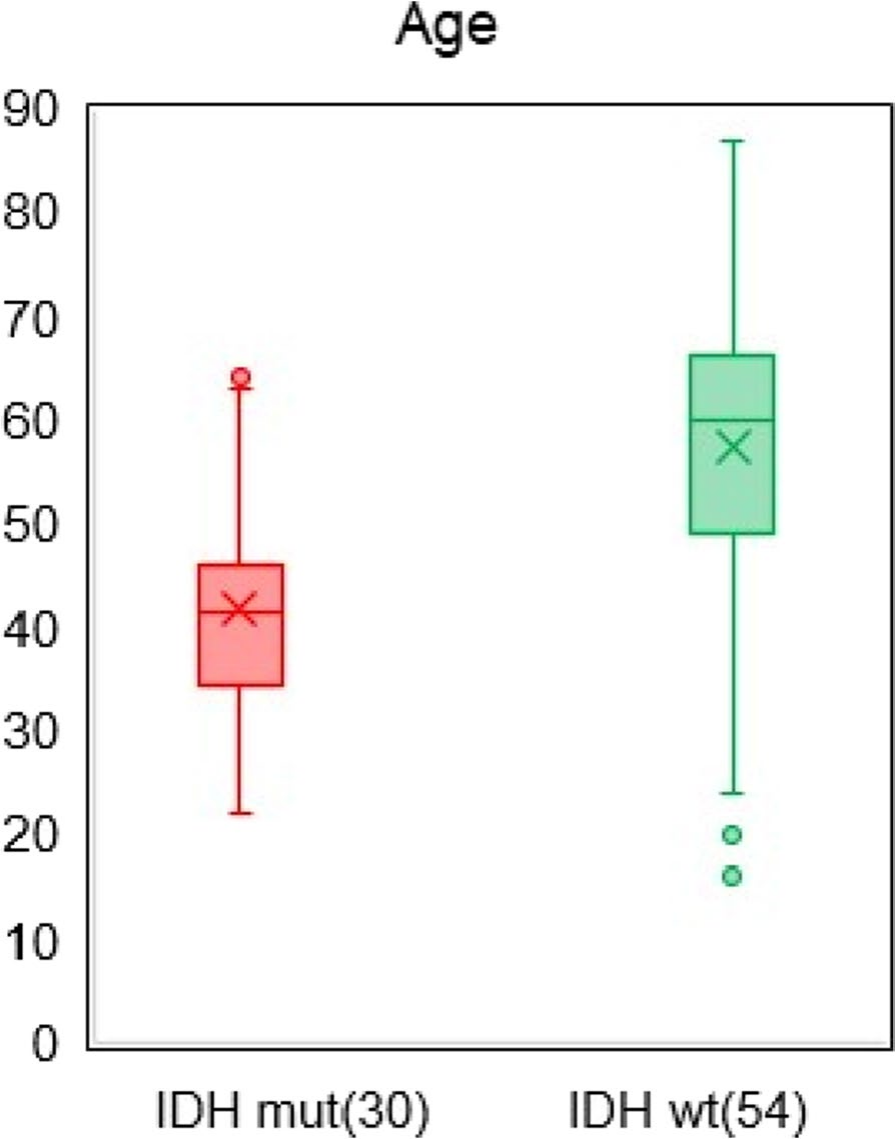
Age distribution of 30 patients with IDH-mutant glioma and 54 patients with IDH wild-type glioma

Many studies have reported the neurotoxic effects of polymerized amyloid on brain function and the enhancement of inflammation by promoting glial activity [59, 67–71]. Why, then, was a greater tendency for protein aggregation observed in the plasma of the IDH variant, which is considered a low-grade glioma? The number of amyloid aggregates deposited in the brain is known to increase with age, but the age distribution of the patients in this study (Fig. 11) shows that the patients in the IDH mutant group were younger than those in the IDH wild-type group, which may have negated the effect of age. IDH plays an important role in maintaining cellular homeostasis in tumor cells, and IDH mutations cause metabolic reprogramming and induce multiple epigenetic changes [66]. The microtubule-associated protein TAU is highly expressed in IDH-mutant gliomas, which is detrimental to gliomas without EGFR mutations because it inhibits angiogenesis [72, 73]. TAU itself is highly aggregative, and as its concentration increases, its seeding (release into the extracellular space) and diffusion (reuptake by receptor neurons) are repeated, accelerating the formation of higher order aggregates with amyloid-β and other compounds [74–78]. This may be at least one of the reasons why the formation of neurotoxic amyloid was more pronounced in the IDH-variant, which is considered low-grade. Although spectroscopy alone cannot identify the content of amyloid aggregates, the almost complete disappearance of the peak at 1540 cm^−1^, which is the N–H absorption maximum of the helical peptide, indicates that amyloid-like aggregation of blood proteins is advanced, especially in IDH-mutant gliomas.

Identification of the IDH mutation status revealed various obvious signs in the IR spectra of plasma from dozens of cases. A tendency toward amyloid aggregation was observed in the plasma of patients with IDH-mutant gliomas before the treatment. The causal relationship between this phenomenon and the formation and progression of IDH-mutant gliomas is currently unknown. Blood analysis reduces the burden on the patient and allows for multiple testing while ensuring real-time results that reflect the patient's general condition at the time of blood collection. With this advantage, we hope to continue our efforts to elucidate the mechanism underlying glioma pathogenesis by conducting follow-up studies in the same patients.

The results of the present study are promising because they indicate that peripheral blood can provide comparable data to tissue samples regarding the development and molecular genetic classification of gliomas that arise in the brain, which is a region with limited access. However, some limitations of this approach must be addressed. First, the plasma samples in this study were collected at a single medical institution, and the ethnicity of the patients was limited. Statistical results, including multivariate analysis, were obtained from a dataset of 156 specimens. The number of IDH-mutant glioma cases was low (30) in distinguishing the presence or absence of IDH mutation. This is due to the rarity of the IDH mutation in glioma [79], making it difficult to secure enough cases. Therefore, it is important to note that our results are based on a limited number of assessed cases. Next, the FTIR-ATR method was used to measure the IR absorption spectrum of plasma, but this measurement system, in which the mid-infrared light is derived from the FTIR and incident on the multi-reflectance IRE, is not a popular method due to the difficulty of alignment and the price of bench-top FTIR and IRE. Hence, the construction of a measurement system that ensures the same level of measurement accuracy and versatility as the present one must be considered, starting with the IRE part. The identification of disease from the IR absorption spectrum of plasma and subsequent molecular genetic classification is also based on the premise that it is used in combination with other information, such as diagnostic imaging. Cross-validation using 30% of the test data for IDH mutation status identification from plasma spectra yielded an accuracy of 75%; this discrimination rate also indicates the limitations of using this information alone. Although the molecular genetic classification of gliomas has been established with great effort over the past several decades, the numerous combinations of genetic mutations, levels of post-translational modifications, and biological responses associated with each mutation are varied and ever-changing. Therefore, discrimination using spectral analysis of blood components reflecting these factors is only the result of the superposition of individual probabilities and exceptions are always possible.

## Conclusion

To establish a less invasive method for glioma identification and prognosis prediction, we distinguished gliomas based on multivariate analysis of the mid-IR absorption spectra of plasma and determined the presence of IDH mutations in patients. FTIR-ATR spectra of plasma were obtained from 84 pretreatment patients and 72 healthy persons, and PLS discriminant analysis was performed using the absorbance of each wavenumber in the fingerprint region of biomolecules as an explanatory variable. A high accuracy of 89% (AUC of ROC curve = 0.957) was obtained through one-off cross-validation and 83% (AUC = 0.908) by cross-validation against 30% test data for the distinguishing of patients from non-patients. Cross-validation of IDH mutations within the patient group yielded an accuracy of 86% (AUC = 0.881) for one-off cross-validation and 75% (AUC = 0.752) for cross-validation against 30% test data. The IR absorption spectra of peripheral blood demonstrated the potential for distinguishing glioma disease and for molecular genetic classification. Absorption spectral characteristics showed that patients with glioma presented significant changes in the conformational composition of blood proteins, which were more pronounced in patients with IDH mutations. The proportion of β-sheet structures in blood proteins was significantly high in patients with glioma, and the characteristics of amyloid aggregation were obvious in patients with IDH mutations. This observation in peripheral blood may serve as a biomarker in minimally invasive liquid biopsy and may contribute to prognostic prediction of glioma and target setting for treatment and drug discovery. In the future, we aim to investigate a larger cohort of patients to establish more precise diagnostic criteria for use in clinical practice.

## Data Availability

No datasets were generated or analysed during the current study.

## Abbreviations

IDH: Isocitrate dehydrogenase
DNA: Deoxyribonucleic acid
MR: Magnetic resonance
2-HG: 2-Hydroxyglutarate
FTIR: Fourier transform infrared
MIR: Mid-infrared
EDTA: Ethylenediaminetetraacetic acid
ATR: Attenuated total reflection
IRE: Internal reflection element
PLS-DA: Partial least squares discriminant analysis
ROC: Receiver operating characteristic
AUC: Area under the curve
LOO: Leave-one-out
VIP: Variables importance of projection
Aβ: Amyloid beta
EGFR: Epidermal growth factor receptor

## References

[1] Solomou G, Finch A, Asghar A, Bardella C (2023). Mutant IDH in gliomas: Role in cancer and treatment options. Cancers.

[2] Alzial G, Renoult O, Paris F, Gratas C, Clavreul A, Pecqueur C (2022). Wild-type isocitrate dehydrogenase under the spotlight in glioblastoma. Oncogene.

[3] Han S, Liu Y, Cai SJ, Qian M, Ding J, Larion M (2020). IDH mutation in glioma: molecular mechanisms and potential therapeutic targets. Br J Cancer.

[4] Al-Khallaf H (2017). Isocitrate dehydrogenases in physiology and cancer: biochemical and molecular insight. Cell Biosci.

[5] Suzuki H, Aoki K, Chiba K, Sato Y, Shiozawa Y, Shiraishi Y (2015). Mutational landscape and clonal architecture in grade II and III gliomas. Nat Genet.

[6] Brat DJ, Verhaak RG, Aldape KD, Yung WK, Salama SR, Cancer Genome Atlas Research Network (2015). Comprehensive, Integrative genomic analysis of diffuse lower-grade gliomas. N Engl J Med.

[7] Dang L, White DW, Gross S, Bennett BD, Bittinger MA, Driggers EM (2009). Cancer-associated IDH1 mutations produce 2-hydroxyglutarate. Nature.

[8] Parsons DW, Jones S, Zhang X, Lin JC, Leary RJ, Angenendt P (2008). An integrated genomic analysis of human glioblastoma multiforme. Science.

[9] Louis DN, Perry A, Wesseling P, Brat DJ, Cree IA, Figarella-Branger D (2021). The 2021 WHO classification of tumors of the central nervous system: a summary. Neuro Oncol.

[10] Kawaguchi T, Sonoda Y, Shibahara I, Saito R, Kanamori M, Kumabe T (2016). Impact of gross total resection in patients with WHO grade III glioma harboring the IDH 1/2 mutation without the 1p/19q co-deletion. J Neurooncol.

[11] Sanai N, Polley MY, McDermott MW, Parsa AT, Berger MS (2011). An extent of resection threshold for newly diagnosed glioblastomas. J Neurosurg.

[12] Choi C, Ganji SK, DeBerardinis RJ, Hatanpaa KJ, Rakheja D, Kovacs Z (2012). 2-hydroxyglutarate detection by magnetic resonance spectroscopy in IDH-mutated patients with gliomas. Nat Med.

[13] Bhandari AP, Liong R, Koppen J, Murthy SV, Lasocki A (2021). Noninvasive determination of *IDH* and 1p19q status of lower-grade gliomas using MRI radiomics: A systematic review. AJNR Am J Neuroradiol.

[14] Kanamori M, Maekawa M, Shibahara I, Saito R, Chonan M, Shimada M (2018). Rapid detection of mutation in isocitrate dehydrogenase 1 and 2 genes using mass spectrometry. Brain Tumor Pathol.

[15] Ray A, Vohra TK (2022). Liquid biopsy—from bench to bedside. Neurooncol Adv.

[16] Ghorbani A, Avery LM, Sohaei D, Soosaipillai A, Richer M, Horbinski C (2023). Discovery of novel glioma serum biomarkers by proximity extension assay. Clin Proteom.

[17] Goutnik M, Lucke-Wold B (2022). Commentary: Evaluating potential glioma serum biomarkers, with future applications. World J Clin Oncol.

[18] Sharma G, Jain SK, Sinha VD (2021). Peripheral inflammatory blood markers in diagnosis of glioma and IDH status. J Neurosci Rural Pract.

[19] Ali H, Harting R, de Vries R, Ali M, Wurdinger T, Best MG (2021). Blood-based biomarkers for glioma in the context of gliomagenesis: A systematic review. Front Oncol.

[20] Cabezas-Camarero S, García-Barberán V, Pérez-Alfayate R, Casado-Fariñas I, Sloane H, Jones FS (2022). Detection of IDH1 mutations in plasma using BEAMing technology in patients with gliomas. Cancers (Basel).

[21] Zhang S, Zhang J, Hu X, Yin S, Yuan Y, Xia L (2023). Noninvasive detection of brain gliomas using plasma cell-free DNA 5-hydroxymethylcytosine sequencing. Int J Cancer.

[22] Voronina L, Leonardo C, Mueller-Reif JB, Geyer PE, Huber M, Trubetskov M (2021). Molecular origin of blood-based infrared spectroscopic ffingerprints*. Angew Chem Int Ed Engl.

[23] Kochan K, Bedolla DE, Perez-Guaita D, Adegoke JA,Chakkumpulakkal Puthan Veettil T, Martin M,  (2021). Infrared spectroscopy of blood. Appl Spectrosc.

[24] Huber M, Kepesidis KV, Voronina L, Fleischmann F, Fill E, Hermann J (2021). Infrared molecular fingerprinting of blood-based liquid biopsies for the detection of cancer. Elife.

[25] Su KY, Lee WL (2020). Fourier transform infrared spectroscopy as a cancer screening and diagnostic tool: A review and prospects. Cancers.

[26] Roy S, Perez-Guaita D, Andrew DW, Richards JS, McNaughton D, Heraud P (2017). Simultaneous ATR-FTIR based determination of malaria parasitemia, glucose and urea in whole blood dried onto a glass slide. Anal Chem.

[27] Roy S, Perez-Guaita D, Bowden S, Heraud P, Wood BR (2019). Spectroscopy goes viral: Diagnosis of hepatitis B and C virus infection from human sera using ATR-FTIR spectroscopy. Clin Spectrosc.

[28] Sitole L, Steffens F, Krüger TP, Meyer D (2014). Mid-ATR-FTIR spectroscopic profiling of HIV/AIDS sera for novel systems diagnostics in global health. OMICS.

[29] Wang X, Shen X, Sheng D, Chen X, Liu X (2014). FTIR spectroscopic comparison of serum from lung cancer patients and healthy persons. Spectrochim Acta A Mol Biomol Spectrosc.

[30] Theakstone AG, Brennan PM, Jenkinson MD, Goodacre R, Baker MJ (2023). Investigating centrifugal filtration of serum-based FTIR spectroscopy for the stratification of brain tumours. PLoS ONE.

[31] Cameron JM, Brennan PM, Antoniou G, Butler HJ, Christie L, Conn JJA (2022). Clinical validation of a spectroscopic liquid biopsy for earlier detection of brain cancer. Neurooncol Adv.

[32] Theakstone AG, Brennan PM, Jenkinson MD, Mills SJ, Syed K, Rinaldi C (2021). Rapid spectroscopic liquid biopsy for the universal detection of brain tumours. Cancers.

[33] Brennan PM, Butler HJ, Christie L, Hegarty MG, Jenkinson MD, Keerie C (2021). Early diagnosis of brain tumors using a novel spectroscopic liquid biopsy. Brain Commun.

[34] Cameron JM, Butler HJ, Smith BR, Hegarty MG, Jenkinson MD, Syed K (2019). Developing infrared spectroscopic detection for stratifying brain tumour patients: glioblastoma multiforme vs. lymphoma. Analyst.

[35] Butler HJ, Brennan PM, Cameron JM, Finlayson D, Hegarty MG, Jenkinson MD (2019). Development of high-throughput ATR-FTIR technology for rapid triage of brain cancer. Nat Commun.

[36] Hands JR, Clemens G, Stables R, Ashton K, Brodbelt A, Davis C (2016). Brain tumour differentiation: rapid stratified serum diagnostics via attenuated total reflection Fourier-transform infrared spectroscopy. J Neurooncol.

[37] Chen F, Meng C, Qu H, Cheng C, Chen C, Yang B (2021). Human serum mid-infrared spectroscopy combined with machine learning algorithms for rapid detection of gliomas. Photodiagnosis Photodyn Ther.

[38] Smith BR, Ashton KM, Brodbelt A, Dawson T, Jenkinson MD, Hunt NT (2016). Combining random forest and 2D correlation analysis to identify serum spectral signatures for neuro-oncology. Analyst.

[39] Hands JR, Dorling KM, Abel P, Ashton KM, Brodbelt A, Davis C (2014). Attenuated total reflection Fourier transform infrared (ATR-FTIR) spectral discrimination of brain tumour severity from serum samples. J Biophotonics.

[40] Hands JR, Abel P, Ashton K, Dawson T, Davis C, Lea RW (2013). Investigating the rapid diagnosis of gliomas from serum samples using infrared spectroscopy and cytokine and angiogenesis factors. Anal Bioanal Chem.

[41] Guleken Z, Bulut H, Gültekin Gİ, Arıkan S, Yaylım İ, Hakan MT (2021). Assessment of structural protein expression by FTIR and biochemical assays as biomarkers of metabolites response in gastric and colon cancer. Talanta.

[42] Guo S, Wei G, Chen W, Lei C, Xu C, Guan Y (2022). Fast and deep diagnosis using blood-based ATR-FTIR spectroscopy for digestive tract cancers. Biomolecules.

[43] Kepesidis KV, Bozic-Iven M, Huber M, Abdel-Aziz N, Kullab S, Abdelwarith A (2021). Breast-cancer detection using blood-based infrared molecular fingerprints. BMC Cancer.

[44] Tołpa B, Depciuch J, Jakubczyk P, Paja W, Pancerz K, Wosiak A (2023). Fourier transform infrared spectroscopic marker of glioblastoma obtained from machine learning and changes in the spectra. Photodiagnosis Photodyn Ther.

[45] Iturrioz-Rodríguez N, De Pasquale D, Fiaschi P, Ciofani G (2022). Discrimination of glioma patient-derived cells from healthy astrocytes by exploiting Raman spectroscopy. Spectrochim Acta A Mol Biomol Spectrosc.

[46] Riva M, Sciortino T, Secoli R, D'Amico E, Moccia S, Fernandes B (2021). Glioma biopsies classification using Raman spectroscopy and machine learning models on fresh tissue samples. Cancers (Basel).

[47] Sciortino T, Secoli R, d’Amico E, Moccia S, Conti Nibali M, Gay L (2021). Raman spectroscopy and machine learning for IDH genotyping of unprocessed glioma biopsies. Cancers.

[48] Livermore LJ, Isabelle M, Bell IM, Scott C, Walsby-Tickle J, Gannon J (2019). Rapid intraoperative molecular genetic classification of gliomas using Raman spectroscopy. Neurooncol Adv.

[49] Uckermann O, Juratli TA, Galli R, Conde M, Wiedemuth R, Krex D (2018). Optical analysis of glioma: Fourier-transform infrared spectroscopy reveals the IDH1 mutation status. Clin Cancer Res.

[50] Cameron JM, Conn JJA, Rinaldi C, Sala A, Brennan PM, Jenkinson MD (2020). Interrogation of IDH1 status in gliomas by Fourier transform infrared spectroscopy. Cancers (Basel).

[51] Quesnel A, Coles N, Angione C, Dey P, Polvikoski TM, Outeiro TF (2023). Glycosylation spectral signatures for glioma grade discrimination using Raman spectroscopy. BMC Cancer.

[52] Barker M, Rayens W (2003). Partial least squares for discrimination. J Chemom.

[53] Hajian-Tilaki K (2013). Receiver operating characteristic (ROC) curve analysis for medical diagnostic test evaluation. Casp J Intern Med.

[54] Anwardeen NR, Diboun I, Mokrab Y, Althani AA, Elrayess MA (2023). Statistical methods and resources for biomarker discovery using metabolomics. BMC Bioinform.

[55] Wood BR (2016). The importance of hydration and DNA conformation in interpreting infrared spectra of cells and tissues. Chem Soc Rev.

[56] Deleris G, Petibois C (2003). Applications of FT-IR spectrometry to plasma contents analysis and monitoring. Vib Spectrosc.

[57] Lombardi G, Corona G, Bellu L, Della Puppa A, Pambuku A, Fiduccia P (2015). Diagnostic value of plasma and urinary 2-hydroxyglutarate to identify patients with isocitrate dehydrogenase-mutated glioma. Oncologist.

[58] De Meutter J, Goormaghtigh E (2021). Amino acid side chain contribution to protein FTIR spectra: impact on secondary structure evaluation. Eur Biophys J.

[59] Pavliukeviciene B, Zentelyte A, Jankunec M, Valiuliene G, Talaikis M, Navakauskiene R (2019). Amyloid β oligomers inhibit growth of human cancer cells. PLoS ONE.

[60] Goldblatt G, Cilenti L, Matos JO, Lee B, Ciaffone N, Wang QX (2017). Unmodified and pyroglutamylated amyloid β peptides form hypertoxic hetero-oligomers of unique secondary structure. FEBS J.

[61] Sarroukh R, Goormaghtigh E, Ruysschaert JM, Raussens V (2013). ATR-FTIR: a "rejuvenated" tool to investigate amyloid proteins. Biochim Biophys Acta.

[62] Matsubara T, Yasumori H, Ito K, Shimoaka T, Hasegawa T, Sato T (2018). Amyloid-β fibrils assembled on ganglioside-enriched membranes contain both parallel β-sheets and turns. J Biol Chem.

[63] Waeytens J, Mathurin J, Deniset-Besseau A, Arluison V, Bousset L, Rezaei H (2021). Probing amyloid fibril secondary structures by infrared nanospectroscopy: experimental and theoretical considerations. Analyst.

[64] Jin WS, Bu XL, Liu YH, Shen LL, Zhuang ZQ, Jiao SS (2017). Plasma amyloid-beta levels in patients with different types of cancer. Neurotox Res.

[65] Zayas-Santiago A, Díaz-García A, Nuñez-Rodríguez R, Inyushin M (2022). Accumulation of amyloid beta in human glioblastomas. Clin Exp Immunol.

[66] Singh S, Joshi V, Upadhyay A (2023). Amyloids and brain cancer: molecular linkages and crossovers. Biosci Rep.

[67] Ferreira ST, Lourenco MV, Oliveira MM, De Felice FG (2015). Soluble amyloid-β oligomers as synaptotoxins leading to cognitive impairment in Alzheimer's disease. Front Cell Neurosci.

[68] Cho MH, Cho K, Kang HJ, Jeon EY, Kim HS, Kwon HJ (2014). Autophagy in microglia degrades extracellular β-amyloid fibrils and regulates the NLRP3 inflammasome. Autophagy.

[69] Stroud JC, Liu C, Teng PK, Eisenberg D (2012). Toxic fibrillar oligomers of amyloid-β have cross-β structure. Proc Natl Acad Sci USA.

[70] Wang H, Ma J, Tan Y, Wang Z, Sheng C, Chen S (2010). Amyloid-beta1-42 induces reactive oxygen species-mediated autophagic cell death in U87 and SH-SY5Y cells. J Alzheimers Dis.

[71] Emilie C, Rabia S, Shiori TK, Leonid B, Sylvie D, Yves FD (2009). Antiparallel β-sheet: a signature structure of the oligomeric amyloid β-peptide. Biochem J.

[72] Papin S, Paganetti P (2020). Emerging evidences for an Implication of the neurodegeneration-associated protein tau. Cancer Brain Sci.

[73] Gargini R, Segura-Collar B, Herránz B, García-Escudero V, Romero-Bravo A, Núñez FJ (2020). The IDH-TAU-EGFR triad defines the neovascular landscape of diffuse gliomas. Sci Transl Med.

[74] Polanco J, Li C, Bodea LG, Martinez-Marmol R, Meunier FA, Götz J (2018). Amyloid-β and tau complexity – towards improved biomarkers and targeted therapies. Nat Rev Neurol.

[75] Guzman-Velez E, Diez I, Schoemaker D, Pardilla-Delgado E, Vila-Castelar C, Fox-Fuller JT (2022). Amyloid-beta and tau pathologies relate to distinctive brain dysconnectomics in preclinical autosomal-dominant Alzheimer's disease. Proc Natl Acad Sci USA.

[76] Korte N, Nortley R, Attwell D (2020). Cerebral blood flow decrease as an early pathological mechanism in Alzheimer's disease. Acta Neuropathol.

[77] Busche MA, Hyman BT (2020). Synergy between amyloid-β and tau in Alzheimer’s disease. Nat Neurosci.

[78] Bloom GS (2014). Amyloid-β and tau: The trigger and bullet in Alzheimer disease pathogenesis. JAMA Neurol.

[79] Iorgulescu JB, Sun C, Neff C, Cioffi G, Gutierrez C, Kruchko C (2022). Molecular biomarker-defined brain tumors: Epidemiology, validity, and completeness in the United States. Neuro Oncol.

